# Supplementary material for: Mitogenome of the leaf-footed bug Notobitus montanus (Hemiptera: Coreidae) and a phylogenetic analysis of Coreoidea
Source: PLoS One. 2023 Feb 10;18(2):e0281597. doi: 10.1371/journal.pone.0281597 (PMC9916562; doi:10.1371/journal.pone.0281597)
Supplement: S1 Table — (DOCX) [file pone.0281597.s004.docx]

**Table S1. List of species for phylogenetic analysis**

| Family | Subfamily | Species | Accession Number |
| --- | --- | --- | --- |
| Stenocephalidae |  | *Dicranocephalus femoralis* | JQ910990 |
| Rhopalidae | Rhopalinae | *Corizus* sp. | KM983397 |
|  |  | *Chorosoma macilentum* | MN412594 |
|  |  | *Aeschyntelus notatus* | NC_012446 |
|  |  | *Stictopleurus subviridis* | NC_012888 |
|  |  | *Myrmus lateralis* | NC_046898 |
|  |  | *Liorhyssus hyalinus* | NC_061753 |
| Alydidae | Alydinae | *Megalotomus costalis* | MZ677327 |
|  |  | *Riptortus pedestris* | NC_012462 |
|  |  | *Daclera levana* | OM489376 |
|  |  | *Camptopus lateralis* | OM489377 |
|  |  | *Melanacanthus marginatus* | OM489378 |
|  | Micrelytrinae | *Leptocorisa lepida* | OL697751 |
|  |  | *Planusocoris schaeferi* | OL702783 |
|  |  | *Grypocephalus pallipectus* | OL702784 |
|  |  | *Paramarcius puncticeps* | OM489375 |
| Coreidae | Pseudophloeinae | *Clavigralla tomentosicollis* | KY274846 |
|  | Hydarinae | *Hydaropsis longirostris* | NC_012456 |
|  | Coreinae | *Enoplops potanini* | NC_046833 |
|  |  | *Cletus punctiger* | NC_050997 |
|  |  | *Acanthocoris* sp. FS-2019 | MF497707 |
|  |  | *Notobitus montanus* | ON052831 |
|  |  | *Cloresmus pulchellus* | NC_042806 |
|  |  | *Leptoglossus membranaceus* | NC_042809 |
|  |  | *Anoplocnemis curvipes* | NC_035509 |
|  |  | *Pseudomictis brevicornis* | NC_042814 |
|  |  | *Mictis tenebrosa* | NC_042811 |
|  |  | *Notopteryx soror* | NC_037376 |
|  |  | *Cletomorpha raja* | MW619725 |
|  |  | *Manocoreus* sp. | MW619724 |
|  |  | *Molipteryx lunata* | NC_042807 |
| （Outgroup） |  | *Malcus inconspicuus* | NC_012458 |
|  |  | *Physopelta gutta* | NC_012432 |
|  |  | *Nezara viridula* | NC_011755 |
