# Supplementary material for: Mitogenome of the leaf-footed bug Notobitus montanus (Hemiptera: Coreidae) and a phylogenetic analysis of Coreoidea
Source: PLoS One. 2023 Feb 10;18(2):e0281597. doi: 10.1371/journal.pone.0281597 (PMC9916562; doi:10.1371/journal.pone.0281597)
Supplement: S2 Table — (DOCX) [file pone.0281597.s005.docx]

**Table S2. Annotation of the *Notobitus montanus* Mitogenome**

| Gene | Strand | Start | Stop | Length | Intergenic nucleotide | Initiation codon | Stop codon |
| --- | --- | --- | --- | --- | --- | --- | --- |
| *trnI* | J | 1 | 63 | 63 | -3 |  |  |
| *trnQ* | N | 61 | 129 | 69 | 1 |  |  |
| *trnM* | J | 131 | 199 | 69 | 1 |  |  |
| *nad2* | J | 201 | 1200 | 1000 | 0 | ATG | T |
| *trnW* | J | 1201 | 1264 | 64 | -8 |  |  |
| *trnC* | N | 1257 | 1319 | 63 | 0 |  |  |
| *trnY* | N | 1320 | 1382 | 63 | 1 |  |  |
| *cox1* | J | 1384 | 2917 | 1534 | 0 | TTG | T |
| *trnL2* | J | 2918 | 2984 | 67 | 0 |  |  |
| *cox2* | J | 2985 | 3663 | 679 | 0 | ATC | T |
| *trnK* | J | 3664 | 3738 | 75 | 0 |  |  |
| *trnD* | J | 3739 | 3801 | 63 | 0 |  |  |
| *atp8* | J | 3802 | 3963 | 162 | -7 | ATA | TAA |
| *atp6* | J | 3957 | 4628 | 672 | 0 | ATG | TAA |
| *cox3* | J | 4629 | 5415 | 787 | 0 | ATG | T |
| *trnG* | J | 5416 | 5478 | 63 | 0 |  |  |
| *nad3* | J | 5479 | 5831 | 353 | 0 | ATA | TA |
| *trnA* | J | 5832 | 5894 | 63 | 0 |  |  |
| *trnR* | J | 5895 | 5958 | 64 | 0 |  |  |
| *trnN* | J | 5959 | 6024 | 66 | -1 |  |  |
| *trnS1* | J | 6024 | 6093 | 70 | -1 |  |  |
| *trnE* | J | 6093 | 6157 | 65 | 0 |  |  |
| *trnF* | N | 6158 | 6222 | 65 | 4 |  |  |
| *nad5* | N | 6227 | 7939 | 1713 | 1 | ATG | TAA |
| *trnH* | N | 7941 | 8004 | 64 | -1 |  |  |
| *nad4* | N | 8004 | 9320 | 1317 | -7 | ATG | TAA |
| *nad4l* | N | 9314 | 9604 | 291 | 2 | ATT | TAA |
| *trnT* | J | 9607 | 9669 | 63 | 0 |  |  |
| *trnP* | N | 9670 | 9732 | 63 | 8 |  |  |
| *nad6* | J | 9741 | 10219 | 479 | 0 | ATA | TA |
| *cytb* | J | 10220 | 11354 | 1135 | 0 | ATG | T |
| *trnS2* | J | 11355 | 11423 | 69 | 21 |  |  |
| *nad1* | N | 11445 | 12369 | 925 | 0 | ATT | T |
| *trnL1* | N | 12370 | 12434 | 65 | 0 |  |  |
| *rrnL* | N | 12435 | 13715 | 1281 | 0 |  |  |
| *trnV* | N | 13716 | 13783 | 68 | 0 |  |  |
| *rrnS* | N | 13784 | 14567 | 784 | 0 |  |  |
| D-loop | J | 14568 | 16209 | 1642 | 0 |  |  |
