# Supplementary material for: Mitogenome of the leaf-footed bug Notobitus montanus (Hemiptera: Coreidae) and a phylogenetic analysis of Coreoidea
Source: PLoS One. 2023 Feb 10;18(2):e0281597. doi: 10.1371/journal.pone.0281597 (PMC9916562; doi:10.1371/journal.pone.0281597)
Supplement: S3 Table — (DOCX) [file pone.0281597.s006.docx]

**Table S3.** Nucleotide composition of *Notobitus montanus* (%)

| Region | A% | C% | G% | T% | A+T% | G+C% | AT skew | GC skew |
| --- | --- | --- | --- | --- | --- | --- | --- | --- |
| atp6 | 38.84 | 17.11 | 9.97 | 34.08 | 72.92 | 27.08 | 0.065 | -0.264 |
| atp8 | 49.38 | 11.73 | 6.17 | 32.72 | 82.10 | 17.90 | 0.203 | -0.310 |
| cox1 | 33.12 | 17.08 | 15.25 | 34.55 | 67.67 | 32.33 | -0.021 | -0.056 |
| cox2 | 37.41 | 17.53 | 13.70 | 31.37 | 68.78 | 31.22 | 0.088 | -0.123 |
| cox3 | 36.21 | 16.90 | 14.49 | 32.40 | 68.61 | 31.39 | 0.056 | -0.077 |
| cytb | 36.48 | 18.50 | 13.13 | 31.89 | 68.37 | 31.63 | 0.067 | -0.170 |
| nad1 | 22.81 | 8.43 | 16.86 | 51.89 | 74.70 | 25.30 | -0.389 | 0.333 |
| nad2 | 40.00 | 12.90 | 9.30 | 37.80 | 77.80 | 22.20 | 0.028 | -0.162 |
| nad3 | 35.98 | 17.85 | 10.76 | 35.41 | 71.39 | 28.61 | 0.008 | -0.248 |
| nad4 | 24.15 | 9.19 | 14.43 | 52.24 | 76.39 | 23.61 | -0.368 | 0.222 |
| nad5 | 24.75 | 9.22 | 16.17 | 49.85 | 74.61 | 25.39 | -0.336 | 0.274 |
| nad6 | 42.59 | 14.82 | 7.93 | 34.66 | 77.24 | 22.76 | 0.103 | -0.303 |
| nd4L | 23.02 | 6.53 | 17.18 | 53.26 | 76.29 | 23.71 | -0.396 | 0.449 |
| rrnL | 46.76 | 15.46 | 8.43 | 29.35 | 76.11 | 23.89 | 0.229 | -0.294 |
| rrnS | 44.39 | 16.84 | 8.42 | 30.36 | 74.74 | 25.26 | 0.188 | -0.333 |
| trnA | 39.68 | 12.70 | 15.87 | 31.75 | 71.43 | 28.57 | 0.111 | 0.111 |
| trnC | 39.68 | 15.87 | 7.94 | 36.51 | 76.19 | 23.81 | 0.042 | -0.333 |
| trnD | 47.62 | 7.94 | 9.52 | 34.92 | 82.54 | 17.46 | 0.154 | 0.091 |
| trnE | 41.54 | 10.77 | 7.69 | 40.00 | 81.54 | 18.46 | 0.019 | -0.167 |
| trnF | 38.46 | 13.85 | 10.77 | 36.92 | 75.38 | 24.62 | 0.020 | -0.125 |
| trnG | 46.03 | 11.11 | 9.52 | 33.33 | 79.37 | 20.63 | 0.160 | -0.077 |
| trnH | 45.31 | 18.75 | 4.69 | 31.25 | 76.56 | 23.44 | 0.184 | -0.600 |
| trnI | 38.10 | 9.52 | 19.05 | 33.33 | 71.43 | 28.57 | 0.067 | 0.333 |
| trnK | 40.00 | 14.67 | 14.67 | 30.67 | 70.67 | 29.33 | 0.132 | 0.000 |
| trnL1 | 43.08 | 15.38 | 4.62 | 36.92 | 80.00 | 20.00 | 0.077 | -0.538 |
| trnL2 | 38.81 | 10.45 | 14.93 | 35.82 | 74.63 | 25.37 | 0.040 | 0.176 |
| trnM | 37.68 | 15.94 | 11.59 | 34.78 | 72.46 | 27.54 | 0.040 | -0.158 |
| trnN | 42.42 | 9.09 | 12.12 | 36.36 | 78.79 | 21.21 | 0.077 | 0.143 |
| trnP | 39.68 | 15.87 | 6.35 | 38.10 | 77.78 | 22.22 | 0.020 | -0.429 |
| trnQ | 42.03 | 15.94 | 5.80 | 36.23 | 78.26 | 21.74 | 0.074 | -0.467 |
| trnR | 42.19 | 15.63 | 12.50 | 29.69 | 71.88 | 28.13 | 0.174 | -0.111 |
| trnS1 | 32.86 | 17.14 | 20.00 | 30.00 | 62.86 | 37.14 | 0.045 | 0.077 |
| trnS2 | 40.58 | 10.14 | 11.59 | 37.68 | 78.26 | 21.74 | 0.037 | 0.067 |
| trnT | 39.68 | 9.52 | 12.70 | 38.10 | 77.78 | 22.22 | 0.020 | 0.143 |
| trnV | 41.18 | 17.65 | 11.76 | 29.41 | 70.59 | 29.41 | 0.167 | -0.200 |
| trnW | 40.63 | 10.94 | 10.94 | 37.50 | 78.13 | 21.88 | 0.040 | 0.000 |
| trnY | 38.10 | 17.46 | 9.52 | 34.92 | 73.02 | 26.98 | 0.043 | -0.294 |
| Control region | 36.30 | 21.80 | 11.02 | 30.88 | 67.17 | 32.83 | 0.081 | -0.328 |
| Whole genome | 42.26 | 16.54 | 10.65 | 30.54 | 72.81 | 27.19 | 0.161 | -0.216 |
