# Supplementary material for: Mitogenome of the leaf-footed bug Notobitus montanus (Hemiptera: Coreidae) and a phylogenetic analysis of Coreoidea
Source: PLoS One. 2023 Feb 10;18(2):e0281597. doi: 10.1371/journal.pone.0281597 (PMC9916562; doi:10.1371/journal.pone.0281597)
Supplement: S4 Table — (DOCX) [file pone.0281597.s007.docx]

**Table S4. Codon usage in the mitochondrial genome of *Notobitus montanus***

| Codon | Count | RSCU | Codon | Count | RSCU | Codon | Count | RSCU | Codon | Count | RSCU |
| --- | --- | --- | --- | --- | --- | --- | --- | --- | --- | --- | --- |
| UUU(F) | 251 | 1.57 | UCU(S) | 97 | 1.98 | UAU(Y) | 192 | 1.54 | UGU(C) | 44 | 1.57 |
| UUC(F) | 68 | 0.43 | UCC(S) | 24 | 0.49 | UAC(Y) | 57 | 0.46 | UGC(C) | 12 | 0.43 |
| UUA(L) | 264 | 3.36 | UCA(S) | 69 | 1.41 | UAA(*) | 67 | 1.29 | UGA(W) | 56 | 1.23 |
| UUG(L) | 91 | 1.16 | UCG(S) | 16 | 0.33 | UAG(*) | 37 | 0.71 | UGG(W) | 35 | 0.77 |
| CUU(L) | 56 | 0.71 | CCU(P) | 55 | 1.91 | CAU(H) | 74 | 1.53 | CGU(R) | 19 | 1.85 |
| CUC(L) | 11 | 0.14 | CCC(P) | 21 | 0.73 | CAC(H) | 23 | 0.47 | CGC(R) | 3 | 0.29 |
| CUA(L) | 38 | 0.48 | CCA(P) | 32 | 1.11 | CAA(Q) | 47 | 1.24 | CGA(R) | 15 | 1.46 |
| CUG(L) | 12 | 0.15 | CCG(P) | 7 | 0.24 | CAG(Q) | 29 | 0.76 | CGG(R) | 4 | 0.39 |
| AUU(I) | 243 | 1.53 | ACU(T) | 67 | 1.45 | AAU(N) | 175 | 1.59 | AGU(S) | 51 | 1.04 |
| AUC(I) | 74 | 0.47 | ACC(T) | 30 | 0.65 | AAC(N) | 45 | 0.41 | AGC(S) | 36 | 0.74 |
| AUA(M) | 204 | 1.52 | ACA(T) | 76 | 1.64 | AAA(K) | 101 | 1.47 | AGA(S) | 67 | 1.37 |
| AUG(M) | 65 | 0.48 | ACG(T) | 12 | 0.26 | AAG(K) | 36 | 0.53 | AGG(S) | 31 | 0.63 |
| GUU(V) | 92 | 2.36 | GCU(A) | 45 | 1.94 | GAU(D) | 49 | 1.66 | GGU(G) | 64 | 1.77 |
| GUC(V) | 9 | 0.23 | GCC(A) | 16 | 0.69 | GAC(D) | 10 | 0.34 | GGC(G) | 13 | 0.36 |
| GUA(V) | 47 | 1.21 | GCA(A) | 30 | 1.29 | GAA(E) | 58 | 1.29 | GGA(G) | 37 | 1.02 |
| GUG(V) | 8 | 0.21 | GCG(A) | 2 | 0.09 | GAG(E) | 32 | 0.71 | GGG(G) | 31 | 0.86 |

Asterisk (*) indicates STOP codon.
