# Supplementary material for: Mitogenome of the leaf-footed bug Notobitus montanus (Hemiptera: Coreidae) and a phylogenetic analysis of Coreoidea
Source: PLoS One. 2023 Feb 10;18(2):e0281597. doi: 10.1371/journal.pone.0281597 (PMC9916562; doi:10.1371/journal.pone.0281597)
Supplement: Attachment — Submitted filename: Response to Reviewers.docx [file pone.0281597.s008.docx]

Dear Editor,

We appreciate you for your organizing the review for our manuscript (PONE-D-22-21262) entitled " **Mitogenome of *Notobitus montanus* (Hemiptera: Coreidae) and a phylogenetic analysis of Coreoidea**". We have carefully read the comments from the editors and reviewers. The comments and suggestions are very valuable for us to improve this manuscript.

**Rebuttal letter to the editors and reviewers (original comments from the editors and reviewers are in blue, and our responses are in black)**

Comment 1: Please ensure that your manuscript meets PLOS ONE's style requirements, including those for file naming. The PLOS ONE style templates can be found at https://journals.plos.org/plosone/s/file?id=wjVg/PLOSOne_formatting_sample_main_body.pdf and <https://journals.plos.org/plosone/s/file?id=ba62/PLOSOne_formatting_sample_title_authors_affiliations.pdf>

Response: we prepared our manuscript according to the PLOS ONE's style requirements.

Comment 2: We note that the grant information you provided in the ‘Funding Information’ and ‘Financial Disclosure’ sections do not match. When you resubmit, please ensure that you provide the correct grant numbers for the awards you received for your study in the ‘Funding Information’ section.

Response: we have checked the Funding Information and provided the correct numbers.

Comment 3: Thank you for stating the following financial disclosure: "This research was funded by National Natural Science Foundation of China, grant number 32060124; Guizhou Normal University, grant number Qianshixinmiao[2021]A11"

Please state what role the funders took in the study.  If the funders had no role, please state: ""The funders had no role in study design, data collection and analysis, decision to publish, or preparation of the manuscript."" If this statement is not correct you must amend it as needed. Please include this amended Role of Funder statement in your cover letter; we will change the online submission form on your behalf.

Response: we added the sentence (The funders had no role in study design, data collection and analysis, decision to publish, or preparation of the manuscript) in the manuscript.

Comment 4: Thank you for stating the following in your Competing Interests section:  "The authors declare no conflicts of interest."

Please complete your Competing Interests on the online submission form to state any Competing Interests. If you have no competing interests, please state ""The authors have declared that no competing interests exist."", as detailed online in our guide for authors at http://journals.plos.org/plosone/s/submit-now. This information should be included in your cover letter; we will change the online submission form on your behalf.

Response: The authors declare no conflicts of interest. we added the information in online system and cover letter.

Comment 5: Please include your full ethics statement in the ‘Methods’ section of your manuscript file. In your statement, please include the full name of the IRB or ethics committee who approved or waived your study, as well as whether or not you obtained informed written or verbal consent. If consent was waived for your study, please include this information in your statement as well.

Response: we added ethics statement in the ‘Methods’ section. *Notobitus* *montanus* is a common pest of bamboos in China as well as not recorded in the species list-of-ethics committees for research involving animals of the Guizhou Normal University. Therefore, no ethical approval or other relevant permission can be provided for the study.

Comment 6: Please include captions for your Supporting Information files at the end of your manuscript, and update any in-text citations to match accordingly. Please see our Supporting Information guidelines for more information: <http://journals.plos.org/plosone/s/supporting-information>.

Response: we provided captions for your Supporting Information files at the end of your manuscript and checked in-text citations.

Comment 7: Please review your reference list to ensure that it is complete and correct. If you have cited papers that have been retracted, please include the rationale for doing so in the manuscript text, or remove these references and replace them with relevant current references. Any changes to the reference list should be mentioned in the rebuttal letter that accompanies your revised manuscript. If you need to cite a retracted article, indicate the article’s retracted status in the References list and also include a citation and full reference for the retraction notice.

Response: we checked reference carefully. We added five references as follows:

1. Wang H, Varma, Xu, editors. Insect Pests of Bamboos in Asia: An Illustrated Manual2002.
2. Zhang et al. Chinese economic entomography: Hemiptera (one): Science Press; 1985.
3. Stothard P, Wishart DS. Circular genome visualization and exploration using CGView. Bioinformatics (Oxford, England). 2005;21(4):537-9. Epub 2004/10/14. doi: 10.1093/bioinformatics/bti054. PubMed PMID: 15479716.
4. Chen Q, Tian X, Li Y, Chen Q. Complete mitochondrial genome of Liorhyssus hyalinus (Hemiptera: Rhopalidae). Mitochondrial DNA Part B. 2022;7(8):1438-40. doi: 10.1080/23802359.2022.2107456.
5. Zhao Q, Wang J, Wang MQ, Cai B, Zhang HF, Wei JF. Complete Mitochondrial Genome of Dinorhynchus dybowskyi (Hemiptera: Pentatomidae: Asopinae) and Phylogenetic Analysis of Pentatomomorpha Species. Journal of insect science (Online). 2018;18(2). Epub 2018/05/03. doi: 10.1093/jisesa/iey031. PubMed PMID: 29718506; PubMed Central PMCID: PMCPmc5905379.

We deleted eleven references as follows:

1. Schuh RT, Slater JA. True bugs of the world (Hemiptera: Heteroptera): classification and natural history: Cornell University press; 1995.
2. Emberts Z, St Mary CM, Miller CW. Coreidae (Insecta: Hemiptera) Limb Loss and Autotomy. Annals of the Entomological Society of America. 2016;109(5):678-83. doi: 10.1093/aesa/saw037. PubMed PMID: WOS:000384436900002.
3. Emlen DJ. The Evolution of Animal Weapons. Annual Review of Ecology, Evolution, and Systematics. 2008;39:387-413.
4. Soares Leal W, Ricardo Panizzi A, Carla Niva C. Alarm pheromone system of leaf-footed bugLeptoglossus zonatus (Heteroptera: Coreidae). Journal of Chemical Ecology. 1994;20(5):1209-16.
5. García‐González F, Nüñez Y, Ponz F, Roldán ER, Gomendio M. Sperm competition mechanisms, confidence of paternity, and the evolution of paternal care in the golden egg bug (Phyllomorpha laciniata). Evolution. 2003;57(5):1078-88.
6. Procter DS, Moore AJ, Miller CW. The form of sexual selection arising from male–male competition depends on the presence of females in the social environment. Journal of Evolutionary Biology. 2012;25(5):803-12. doi: <https://doi.org/10.1111/j.1420-9101.2012.02485.x>.
7. Panizzi AR, Schaefer CW. Broad-Headed Bugs (Alydidae). In: Panizzi AR, Grazia J, editors. True Bugs (Heteroptera) of the Neotropics. Dordrecht: Springer Netherlands; 2015. p. 537-47.
8. Sheffield N, Song H, Cameron S, Whiting M. A comparative analysis of mitochondrial genomes in Coleoptera (Arthropoda: Insecta) and genome descriptions of six new beetles. Molecular Biology Evolutionary Bioinformatics. 2008;25(11):2499-509.
9. Sima YH, Chen M, Yao R, Li YP, Liu T, Jin X, et al. The complete mitochondrial genome of the Ailanthus silkmoth, Samia cynthia cynthia (Lepidoptera: Saturniidae). Gene. 2013;526(2):309-17. doi: <https://doi.org/10.1016/j.gene.2013.05.048>.
10. Wang JJ, Li DF, Li H, Yang MF, Dai RH. Structural and phylogenetic implications of the complete mitochondrial genome of Ledra auditura. Scientific Reports. 2019;9(1):1-11.
11. Zhao W, Liu D, Jia Q, Wu X, Zhang H. Characterization of the complete mitochondrial genome of Myrmuslateralis (Heteroptera, Rhopalidae) and its implication for phylogenetic analyses. Zookeys. 2021;1070:13-30. doi: 10.3897/zookeys.1070.72742. PubMed PMID: 34819768.

Comment 8: In several cases authors draw major conclusions without providing data or analysis. Most of this conclusion could also be because authors are not aware of several basic concepts in molecular genetics of mitochondria. Authors should know that, while making any big statement they should provide evidence or arguments based on earlier studies to support the claims.

The first claim authors make is that “Most amino acids preferred to use tRNAs encoded by nuclear genome.” It is unclear how authors came to this conclusion. The tRNAs for all 20 amino acids (including the two separate starting codons for leucine and serine) are present in the mitochondrial genome so why do authors mention that the mitochondria is using nuclear tRNAs. If this conclusion is drawn based on the mismatch between the third codon position in the CDS and tRNA anticodon arm, then authors should know that the third codon position is a wobble position.

Response: thank you for the comment! We made mistakes. RNAs cannot transport from cytoplasm to mitochondrion, as a result, mitochondrion uses its own RNAs for translation. At the same time, the third codon position is a wobble position. We have deleted corresponding sentences.

Comment 9: The second claim authors make is regarding the overlap between the genes. Authors mention, “The presence of polycistron might contribute to generating overlaps between PCGs.” Authors cite reference [32] for this statement; however, the reference does not make any such claim. This statement is an ignorance of basic molecular biology of mitochondrial genome. First, polycistron has nothing to do with overlap in the genes. Most polycistron exists without overlap in the genes. In the case of mitochondria, the mitochondrial genes are generally transcribed as two large precursor polycistronic transcripts. These transcripts are subsequently cleaved to generate individual mRNAs, tRNAs and rRNAs. So polycistron does not explain the overlapping genes because even the genes that do not have overlap are transcribed from polycistronic transcripts.

Response: thank you for the comment! We made mistakes. We have deleted incorrect sentences.

Comment 10: Minor comments: Authors will need to improve manuscript writing substantially.

(1) Authors can add the common name “Leaf-footed bug” before Notobitus montanus to make it more accessible to non technical readers. Suggested title: Mitogenome of the leaf-footed bug Notobitus montanus (Hemiptera: Coreidae) and a phylogenetic analysis of Coreoidea.

Response: thank you for the comment! We have added the common name “Leaf-footed bug” before *Notobitus montanus*. We rewrote the manuscript according to comments.

Comment 11: (2) As per the zoological nomenclatural rules, authority of scientific names that are written as per their original combination are not in parenthesis. Notobitus montanus is the original combination in which the species was described. So the authority and year cannot be in parenthesis. This should be - Notobitus montanus Hsiao, 1963. Make this change in abstract and remaining text.

Response: scientific names have been checked and corrected.

Comment 12: (3) Abstract is not drafted properly. Authors unnecessarily provide details on the start and stop codons of coding genes when most of this is common in other organisms as well. In the manuscript authors talk about RSCU, compare codon usage with other species, they also provide comparison of D-loop of several species but none of this analysis is mentioned in the abstract. An abstract is a vital part of the manuscript and should be presented properly with a proper flow. There should be one or two lines providing the background of the study and its importance. One or two lines describing the methods use (also mention you determined secondary structure of tRNAs, compared genomes for codon usage and performed phylogeny). Important results in a few lines and implications of this study in understanding ecology and evolution of the species as a conclusion.

Response: we rewrote the abstract according the comment as follows:

*Notobitus montanus* Hsiao, 1963 is a major pest of bamboos, however, it is difficult to discriminate *Notobitus* bamboo pests especially their nymphs. To overcome the challenge, the mitogenome of *N. montanus* (ON052831) was decoded using next-generation sequencing. The mitogenome, with 42.26% A, 30.54% T, 16.54% C, and 10.65% G, is 16,209 bp in size. Codon usage analysis indicated that high frequently used codons used either A or T at the third position of the codon. Amino acid usage analysis showed that leucine 2, phenylalanine, isoleucine and tyrosine were the most abundant in 31 Coreoidea species. Thirteen protein-coding genes (PCGs) were evolving under purifying selection, and *nad5* and *cox1* had the lowest and strongest purifying selection stress, respectively. Correlation analysis showed that evolutionary rate had positive correlation with A+T content. No tandem repeat was detected in the non-coding region. The phylogenetic tree showed that Alydidae and Coreidae were not monophyletic. However, the topology of phylogenetic trees, based on 13 PCGs, was not in accordance with that of tree, based on ultraconserved element loci or combination of 13 PCGs and two rRNAs. It seems that their relationships are complex, which need revaluation and revision. The mitogenomic information of *N. montanus* could shed light on the discrimination of *Notobitus* bamboo pests, and evolution of Coreoidea.

Comment 13: (4) The word “taxology” is normally not used in the field. What authors are referring to is called “taxonomy”.

Response: taxology was replaced by taxonomy.

Comment 14: (5) Introduction does not provide any information on the taxa under study Notobitus montanus and why it is important to study its mitogenome.

Response: we added information on *Notobitus montanus*, and why we decoded its mitogenome as follows:

Leaf-footed bugs, hemipteran superfamily Coreoidea, are phytophagous insects which consist of several forest and agricultural pests [1, 2]. For example, *Notobitus montanus* Hsiao, 1963 is a major pest of bamboos, which not only supply human with foods, building materials, crafts, and high-quality paper, but also are involved in landscaping and soil conservation [3]. It distributes in many southern provinces of China, such as Zhejiang, Sichuan, Yunnan, Guizhou, which serves as the pest of serval species of bamboo, including, *Phyllostachys sulphurea* var. Viridis, *Phyllostachys* *glauca*, *Phyllostachys* *heteroclada*, *Phyllostachys* *bambusoides* f. shouzhu, *Phyllostachys heteroclada* var. pubescens, *Pleioblastus amzrus*, *Phyllostachys praecox* f. provernalis [4]. The adults and nymphs of *N. montanus* feed on the sap of bamboo shoot and young bamboo, making bamboo falls into a decline and even dead. A female can lay 20-70 eggs, and the egg hatchability is high. Eggs take up 15-20 days to develop as nymphs, and then nymphs take up 30-50 days to develop as adult, which can live for 330-350 days [4]. Due to its high reproduction, strong ability of flying and gregariousness, *N. montanus* can cause serious economic loss of bamboo [5]. For example, in 1973, 90% of bamboo shoots were attacked by it in Liangping count, Sichuan province, leading 16.4% bamboo shoots dead [4]. Currently, approximately 30 Coreoidea species are recorded as the pest of bamboo. *N. montanus* has similar morphology with *Notobitus meleagris* Fabricius 1787. Especially, it is difficult to discriminate their nymphs [4].

Comment 15: (6) As a rule, authors should use present tense when they are referring to established facts published earlier or any other facts. For example, second last line in first paragraph of results and discussion, “These two overlaps were popular in arthropods [19, 20]” should be “……. overlaps are popular in arthropods…”. Last line in third paragraph of results and discussion, “Incomplete stop codons were usually used in metazoan [18]” should be “Incomplete stop codons are usually used in metazoan [18]”. Similarly, in figure caption of figure 6, “The posterior probabilities were labeled at each node” should be “The posterior probabilities are labeled at each node”.

Response: we have checked the tense carefully and corrected them.

Comment 16: (7) The results are not stated properly and discussed at length. It is actually better to separate results and discussions. Authors can explain all the results in the results section and in the discussion discuss the results with respect to other mitogenomes and also discuss the implications of the results with respect to understanding ecology and evolution of the focal taxa. Authors have provided some analysis with respect to the published genomes of related taxa but the implications of the results are not clear.

Response: the part of results and discussions was divided into two parts (results, discussions). Implications were added.

Comment 17: (8) Figure legends should be self-explanatory and readers should understand the figure from figure legends without referring to the main text. For example, authors use several colours, numbers, etc. in figure 5 but the figure legend does not explain any of these. Similarly, for figure 6, there are two classification schemes shown in the figure but these are not explained in the figure legend. Figure legend of figure 6 also does not explain what data was used for the phylogenetic analysis, both coding and non-coding genes or just the coding genes. Although this is mentioned in the main text, as stated earlier, figure legends should be in details and self-explanatory.

Response: we described the figure legends in details.

Comment 18: (9) Supplementary information should be referred to as Fig. S1, Fig. S2, Table S1, etc. and not S1 Fig., S2 Fig., S1 Table, etc. Also, revise the figure captions to make them self-explanatory.
Response: S1 Fig was replaced by Fig S1. We described the figure captions in details.

Comment 19: Reviewers' comments:

Reviewer #1: Dear Editor,

This manuscript focuses on sequencing a mitogenome of Notobitus montanus (Hemiptera: Coreidae) - the first mitogenome of Notobitus to investigate the mitogenome structure and phylogeny analysis. Their results were consistent with Dong et al. 2022 and didn't get any innovative results but one mitogenome of a species. I have some key points that I will address here if you think it was deserved to publication. Reference

Dong X, Wang K, Tang Z, Zhang Y, Yi W, Xue H, et al. Phylogeny of Coreoidea based on mitochondrial genomes show the paraphyly of Coreidae and Alydidae. Archives of Insect Biochemistry and Physiology. 2022;110(1):e21878.

Response: thank you for the comment! Our results showed that Alydidae and Coreidae were not monophyly (Fig 6), which was in accordance with previous studies [1, 6]. At subfamily level, Alydinae and Coreinae showed monophyly with high support (PP= 1, BS>97). Our phylogenetic tree, based on 13 PCGs, showed that their relationship was (((((Alydinae + Pseudophloeinae) + Hydarinae) + Micrelytrinae) + Coreinae) + Rhopalinae). One study, based on 13 PCGs and 2 rRNAs showed their relationship was ((((Coreinae + Hydarinae) + Micrelytrinae) + (Alydinae + Pseudophloeinae)) + Rhopalinae) (Dong X), Another study, based on ultraconserved element loci showed their relationship was ((((Micrelytrinae + Hydarinae) + (Alydinae + Pseudophloeinae)) + Coreinae) + Rhopalinae). Rhopalinae was a sister group of the remaining five subfamilies in common. In addition, Alydinae had a closer relationship with Pseudophloeinae than the remaining subfamilies. Our tree showed that Coreinae was a sister group of the remaining four subfamilies, including Alydinae, Pseudophloeinae, Hydarinae, Micrelytrinae, which was consistent with the tree based on ultraconserved element loci but not the tree based on 13 PCGs and 2 rRNAs (Dong X). Previous study indicated that the sequence of rRNAs was not suitable for phylogenetic tree reconstruction [34]. However, the topology of our tree was not in accordance with that of tree based on ultraconserved element loci. It seems that their relationships are complex, and their relationships need revaluation and revision [9].

In addition, codon usage, amino acid usage, tRNA secondary structure, and the evolutionary pattern of 13 PCGs, were systematically analyzed in our study, however, Dong et al did not analyzed these contents. Our results as follows:

Codon usage analysis indicated that high frequently used codons used either A or T at the third position of the codon. Amino acid usage analysis showed that leucine 2, phenylalanine, isoleucine and tyrosine were the most abundant in 31 Coreoidea species. Thirteen protein-coding genes (PCGs) were evolving under purifying selection, and *nad5* and *cox1* had the lowest and strongest purifying selection stress, respectively. Correlation analysis showed that evolutionary rate had positive correlation with A+T content. No tandem repeat was detected in the non-coding region.

Comment 20: Comments to Author:

Major questions:

Q1: Coreoidea consists of five extant families, but mitochondrial genomes in your manuscript only covered four families (Hyocephalidae not included). You should state clearly the group used for phylogeny reconstruction in all related description. Such as this sentence “The phylogenetic tree of Coreoidea was constructed with the new mitogenome and 30 mitogenomes extracted from NCBI.”

Response: we added information as follows:

Coreoidea consists of five extant families: Coreidae, Alydidae, Rhopalidae, Stenocephalidae and Hyocephalidae [1, 6, 7]. Currently, no mitogenome of Hyocephalidae species is available in NCBI, as a result, only four families were analyzed in the study.

The mitogenomes of *N. montanus* and 30 available complete mitogenomes of Coreoidea in NCBI were used to construct the phylogenetic tree of Coreoidea (Table S1)

Comment 21: Q2: The methods for genome extraction, sequencing and mitochondrial genome assembly are inaccurate in materials and methods. “DNA was isolated from…. Mitogenomic DNA was fragmented, then ~500 bp DNA was recycled. Paired-end libraries were constructed with the Illumina platform. The mitogenome was sequenced using the…”. It confused me for obtaining the mitogenome between genomic DNA extraction and sequencing or assembly. There is more than one way to get to the mitogenome (sequence genomic DNA and then to isolate mitogenome after sequencing; enrich the mitogenome prior to sequencing and other approach).

Response: we revised methods as follows:

Total DNA was isolated from the muscle tissue of an adult specimen with ONE-4-ALL Genomic DNA Mini-Prep Kit (BS88504, Sangon, Shanghai, China). DNA was fragmented, then ~500 bp DNA was recycled. Paired-end libraries were constructed with the Illumina platform. The DNA was sequenced using the Illumina Hiseq X Ten at the Sangon Biotechnology Company (Shanghai, China). The adapter sequences were removed, and low-quality reads were trimmed with Trimmomatic version 0.36 [17]. The mitogenome of *Cloresmus pulchellus* (NC_042806) was used as reference, and clean reads were assembled with SOAPdenovo2 (version 2.04) [18]. PCGs were identified by BLAST comparison with *C. pulchellus* mitogenome [19].

Comment 22: Q3. In the Transfer RNA and ribosomal RNA genes results, you described the amino acid preferences about the use of tRNAs. “…….preferred to use tRNAs encoded by the mitogenome or nuclear genome”. How do you determine the use in the nuclear genome? And you operate only for Notobitus montanus, cannot represent the whole large group, whether there are relevant references to justify?

Response: we made mistakes; the corresponding sentences were deleted.

Comment 23: Q4: I think that authors should give a detailed and accurate description in results and discussion part about phylogenetic relationship. For example, you cited the reference but inaccurate. “However, based on nucleotide sequences of 13 PCGs and two rRNAs, some studies considered Alydidae and Coreidae were monophyletic [40].” It clarified the sister group of Rhopalidae with Alydidae + Coreidae but not revealed the monophyly of Alydidae and Coreidae because one subfamily of Alydidae were not covered.

Response: we checked all references, and deleted inaccurate descriptions and references.

Comment 24: Minor comments:

Q1. You used many analyses software in materials and methods, but not all of them has corresponding version number. Determine the software version you are using.

Response: software version was added.

Q2. In phylogenetic analysis part, I don’t understand the operation for PCGs in Phylosuite. “These mitogenomes were imported into PhyloSuite. After standardization of sequences, the nucleic acid sequences of 13 PCGs were extracted from these mitogenomes.” I wonder what’s meaning and how to standardize in Phylosuite. That is, what is being done to the sequences.

Response: we added information as follows:

Both trnS and trnL have two tRNAs, however, some submitters of mitogenomes do not discriminate between trnS1 and trnS2, between trnL1 and trnL2. At the same time, one gene may have more than one abbreviation, for example, cytochrome b presents as cytb and cob. In order to solve these problems, all sequences were standardized.

The authors declare no conflicts of interest.

With the above modifications, we think that the revised manuscript has been substantially improved in terms of data presentation and analyses, and appreciate your consideration for its publication in PLOS ONE.

Best regards,

Qianquan Chen, Ph.D.

School of Life Sciences

Guizhou Normal University

E-mail: [qqchen@gznu.edu.cn](mailto:qqchen@gznu.edu.cn)
