# Supplementary material for: Mitogenome of the leaf-footed bug Notobitus montanus (Hemiptera: Coreidae) and a phylogenetic analysis of Coreoidea
Source: PLoS One. 2023 Feb 10;18(2):e0281597. doi: 10.1371/journal.pone.0281597 (PMC9916562; doi:10.1371/journal.pone.0281597)
Supplement: Attachment — Submitted filename: Response to Reviewers.docx [file pone.0281597.s010.docx]

**Rebuttal letter to the editors and reviewers (original comments from the editors and reviewers are in blue, and our responses are in black)**

Comment 1: Discriminate Notobitus from whom? Allied genera or Notobitus montanus from its congeners? Nowhere in your results or discussion you talk about the potential of the genetic data for identifying the species from its close congeners. So why to mention this in the abstract? Keep you study focused on the analysis you have done and the results you have got. The strong point of your study is that you provide first mitogenome characterization for genus Notobitus (although there is data on N. elongatus mitogenome is available it is used only for phylogenetic analysis) and

Response: thank you for the comment and revise!

Comment 2: Discriminate from whom? Your analysis does not have any other species of Notobitus so how can you discriminate different species based on the mitogenome?

Response: the sentence was corrected as “The mitogenomic information of *N. montanus* could shed light on the evolution of Coreoidea”.

Comment 3: This is true. But your analysis does not contain genetic information about any other Notobitus species so how can your study will help in identifying one species from other. Perhaps someone will use your data and use it for genetic delimitation of species in Notobitus. But that is not an achievement of your study. So why to mention it?

Response: thank you for the comment! We deleted the sentence.

Comment 4: Not really. There is mitogenome of N. elongatus also available, although the study only uses it for phylogenetic analysis. So yours is the first characterization of mitogenome of Notobitus. You can mention the other genetic data available from the study by Yi et al. <https://doi.org/10.1111/cla.12501>. Remember that although the paper by Yu et al is in press, the genetic data for complete mitochondria was available from April 2022, before the current manuscript was submitted. So you cannot ignore this available mitogenome.

Response: thank you for the comment! the sentence was corrected as “In this work, the mitogenome of *N. montanus* has been decoded”.

Comment 5: Your comparative analysis is mainly based on PCGs so what difference does this make?

How were the sequences standardized? And what difference does it make if other studies have used different abbreviations? You just take the data for the gene of interest. This is unnecessary and redundant information.

Response: thank you for the comment! The two sentences (Both trnS and trnL have two tRNAs, however, some submitters of mitogenomes do not discriminate between trnS1 and trnS2, between trnL1 and trnL2. At the same time, one gene may have more than one abbreviation, for example, cytochrome b presents as cytb and cob. In order to solve these problems, all sequences were standardized) were deleted.

Comment 6: How are your finding with respect to Yi et al. <https://doi.org/10.1111/cla.12501>, which is based on both mitochondrial and nuclear genes?

Response: the topology of our tree was in accordance with that of tree based on both mitochondrial and nuclear genes. The sentence was revised as “However, the topology of our tree was in accordance with that of tree based on both mitochondrial and nuclear genes but not ultraconserved element loci”. The reference (35.	Ye F, Kment P, Rédei D, Luo J-Y, Wang Y-H, Kuechler SM, et al. Diversification of the phytophagous lineages of true bugs (Insecta: Hemiptera: Heteroptera) shortly after that of the flowering plants. Cladistics. 2022;38(4):403-28. doi: <https://doi.org/10.1111/cla.12501>) has been added.

With the above modifications, we think that the revised manuscript has been substantially improved in terms of data presentation and analyses, and appreciate your consideration for its publication in PLOS ONE.

Best regards,

Qianquan Chen, Ph.D.

School of Life Sciences

Guizhou Normal University

E-mail: [qqchen@gznu.edu.cn](mailto:qqchen@gznu.edu.cn)
